# FibroBox: a novel noninvasive tool for predicting significant liver fibrosis and cirrhosis in HBV infected patients

**DOI:** 10.1186/s40364-020-00215-2

**Published:** 2020-09-25

**Authors:** Xiao-Jie Lu, Xiao-Jun Yang, Jing-Yu Sun, Xin Zhang, Zhao-Xin Yuan, Xiu-Hui Li

**Affiliations:** 1 Department of General Surgery, The First Affiliated Hospital of Nanjing Medical University, Nanjing Medical University, Nanjing, China; 2 Department of Infection, the First Affiliated Hospital of Anhui University of Chinese Medicine, Hefei, China; 3 Department of Medical Imaging, The Fourth People’s Hospital of Huai’an, Huai’an, China; 4 Changchun Medical College, Changchun, Jilin China; 5 Department of Hepatology, Hepatobiliary Disease Hospital of Jilin Province, Changchun, Jilin China; 6 Department of Integrated Traditional Chinese Medicine and Western Medicine, Beijing Youan Hospital, Capital Medical University, Beijing, China

**Keywords:** Liver fibrosis, HBV, Noninvasive diagnosis, Machine learning

## Abstract

**Background:**

China is a highly endemic area of chronic hepatitis B (CHB). The accuracy of existed noninvasive biomarkers including TE, APRI and FIB-4 for staging fibrosis is not high enough in Chinese cohort.

**Methods:**

Using liver biopsy as a gold standard, a novel noninvasive indicator was developed using laboratory tests, ultrasound measurements and liver stiffness measurements with machine learning techniques to predict significant fibrosis and cirrhosis in CHB patients in north and east part of China. We retrospectively evaluated the diagnostic performance of the novel indicator named FibroBox, Fibroscan, aspartate transaminase-to-platelet ratio index (APRI), and fibrosis-4 index (FIB-4) in CHB patients from Jilin and Huai’an (training sets) and also in Anhui and Beijing cohorts (validation sets).

**Results:**

Of 1289 eligible HBV patients who had liver histological data, 63.2% had significant fibrosis and 22.5% had cirrhosis. In LASSO logistic regression and filter methods, fibroscan results, platelet count, alanine transaminase (ALT), prothrombin time (PT), type III procollagen aminoterminal peptide (PIIINP), type IV collagen, laminin, hyaluronic acid (HA) and diameter of spleen vein were finally selected as input variables in FibroBox. Consequently, FibroBox was developed of which the area under the receiver operating characteristic curve (AUROC) was significantly higher than that of TE, APRI and FIB-4 to predicting significant fibrosis and cirrhosis. In the Anhui and Beijing cohort, the AUROC of FibroBox was 0.88 (95% CI, 0.72–0.82) and 0.87 (95% CI, 0.83–0.91) for significant fibrosis and 0.87 (95% CI, 0.82–0.92) and 0.90 (95% CI, 0.85–0.94) for cirrhosis. In the validation cohorts, FibroBox accurately diagnosed 81% of significant fibrosis and 84% of cirrhosis.

**Conclusions:**

FibroBox has a better performance in predicting liver fibrosis in Chinese cohorts with CHB, which may serve as a feasible alternative to liver biopsy.

## Introduction

Hepatitis B virus (HBV) infection has become a major public health threat for its high prevalence (attacking 257 million people worldwide in 2016) [1]. The major complications of CHB include cirrhosis and hepatocellular carcinoma, leading to poor prognosis [2]. Chronic hepatitis B (CHB) is highly endemic in China, with over 74 million hepatitis B surface antigen (HBsAg)-positive patients [2, 3]. The number of CHB patients undergoing antiviral treatment remains uncalculated [4]. To control the spread of CHB in China, it is essential to conduct early diagnosis and intervention of HBV infection.

Fibrosis staging, an approach to assess HBV-induced liver diseases, is efficient to estimate the prognosis of patients and identify those requiring antiviral treatment [5]. Liver biopsy is traditionally recommended as a standard for staging fibrosis [6], but it is restricted with by invasiveness, cost [7, 8], and unavoidable errors from sampling [9, 10]. Therefore, a variety of noninvasive tests have been developed in recent years.

As summarized in EASL-ALEH clinical practice guidelines [11], noninvasive staging usually depends on serum biomarkers-based mathematic calculation and elasticity-based imaging techniques, such as transient elastography (TE) and magnetic resonance elastography (MRE). Although several strategies combining TE and computer algorithm are introduced in the guidelines, they are only applicable for patients infected with hepatitis C virus (HCV). Moreover, no measurements or macro characteristics of imaging methods have been described in strategies.

With machine learning that can tease out the complex, non-linear relationships in the data [12, 13], we conducted a retrospective multicenter study and established a novel multivariate algorithmic model, named FibroBox, in a cohort of CHB patients in Huaian and Jilin, and then evaluated its predictive accuracy in external validation sets from Anhui and Beijing.

## Methods

### Patients

We selected 1843 treatment-naïve CHB patients who underwent liver biopsy, blood test, B-ultrasound examination and Fibroscan (FS402, Echosens, France) at four centers, including Huai’an Fourth People’s Hospital (Huai’an, China) (June 2010–October 2017), Beijing You-An Hospital (Beijing, China) (December 2013–April 2017), Hepatology Hospital of Jilin Province (Jilin, China) (July 2008–October 2016) and The First Affiliated Hospital of Anhui University of Chinese Medicine (Anhui, China) (February 2012–November 2017). Their clinical data were retrospectively collected through hospital information system. Included were those who underwent liver biopsy and at least one of the following criteria: aspartic transaminase (AST) or alanine transaminase (ALT) ≥40 IU/L, liver stiffness ≥6.5 kPa, HBV DNA ≥2000 IU/mL or family history of liver diseases. The exclusion criteria included co-infection with HCV, hepatitis D virus (HDV) or human immunodeficiency virus (HIV), focal hepatic lesion (e.g. HCC, hepatic tuberculosis and any other), significant alcohol intake (> 20 g/day), severe hepatic failure (complications such as jaundice and ascites or transaminases level over 10 times the upper limit of normal (ULN)), acute heart failure and pregnancy and BMI greater than 30 kg/m^2^.

### Liver biopsy

Percutaneous liver biopsy (LB) was performed under the ultrasonic guidance by experienced ultrasonologists. Liver samples were formalin-fixed and paraffin-embedded for subsequent histological analysis. Histological analysis was performed by three senior pathologists in every center. If three different results came from one sample, the consensus was taken as the final decision. Liver samples with less than three portal tracts were considered as poor quality and excluded from the analysis. All the pathologists were blinded to the clinical information. The liver fibrosis was staged by the Metavir system [14]. F ≥ 2 was considered as significant fibrosis and F4 as cirrhosis.

### Transient elastography (Fibroscan)

All liver stiffness measurements (LSMs) were performed using Fibroscan devices (FS402, Echosens, France) by skilled technicians according to the manufacturer’s protocol [15]. The TE results were presented as kilopascal (kPa). For each patient, the median of 10 successfully measured TE values was regarded as the final TE. A measurement was considered invalid if its TE median > 7.1 kPa and interquartile ratio (IQR)/LSM > 0.30 [16].

### Traditional serum index calculation

Aspartate transaminase (AST)-to-platelet ratio index (APRI) [17] and the fibrosis-4 (FIB-4) [18] are two common compound surrogates that use simple formulas to score easily acquired parameters. The formulas of APRI and FIB-4 were shown as follows:

$$ \mathrm{APRI}=\frac{\left(\mathrm{AST}\left(\mathrm{IU}/\mathrm{L}\right)/\mathrm{ULN}\right)\times 100\ }{\mathrm{Platelet}\ \mathrm{count}\ \left({10}^9/\mathrm{L}\right)} $$

$$ \mathrm{FIB}-4=\frac{\mathrm{age}\left(\mathrm{years}\right)\times \mathrm{AST}\left(\mathrm{IU}/\mathrm{L}\right)\kern0.5em }{\mathrm{Platelet}\ \mathrm{count}\ \left({10}^9/\mathrm{L}\right)\times \mathrm{ALT}\left(\mathrm{IU}/\mathrm{L}\right)\hat{\mkern6mu} 1/2} $$

These relevant input parameters were measured when patients were admitted to the hospitals without any interventions.

### Ultrasonic measurement

In this study, the parameters measured during ultrasonic examinations included the size of spleen (mm^2^, length × thickness), the diameter of splenic vein (mm) and the diameter of portal vein (mm). Every parameter was measured for at least three times by experienced ultrasonologists and the mean value was calculated as the final score of each measurement.

### Training sets

Two training data sets of treatment-naïve HBV-infected patients who entirely met the study criteria from Huai’an and Jilin (*n* = 549) were subjected to the algorithmic model (FibroBox). The sets were not absolutely comparable, but the mode could normalize these sets.

### Validation sets

The diagnostic performances of the FibroBox and other noninvasive markers were evaluated with external validation sets from Anhui and Beijing cohorts. In the Anhui (*n* = 408) and Beijing cohorts (*n* = 332), the CHB patients who underwent biopsy with available data on TE, AST, ALT and Platelet count were included in the analysis.

### FibroBox construction

The data characteristics, preprocessing and training/testing procedures of FibroBox were described in Supplement Material 1. All variables were normalized in order to minimize systematic errors from different centers. And then algorithm models (Supplement Material 1) were used to select significant variables and conduct training and validation. The machine learning algorithm was implemented using Python 3.7 (Amsterdam, Netherlands).

### Statistical analysis

The diagnostic accuracy of FibroBox and conventional fibrosis markers (APRI, FIB-4 and Fibroscan) was estimated using the area under the receiver operating characteristic curve (AUROC) and the rate of correctly classified fibrosis/cirrhosis. Delong’s test [19] with a significant level of 0.05 was used to compare AUROC values of the FibroBox and other markers. Agreements between them were described using Cohen’s kappa coefficient. The decision curve analysis (DCA) and ROC analysis were computed with R 3.5.1. Statistical analysis was conducted using SPSS 19.0 (SPSS Inc., Chicago, IL, USA).

## Results

### Study population

**Fig. 1:**
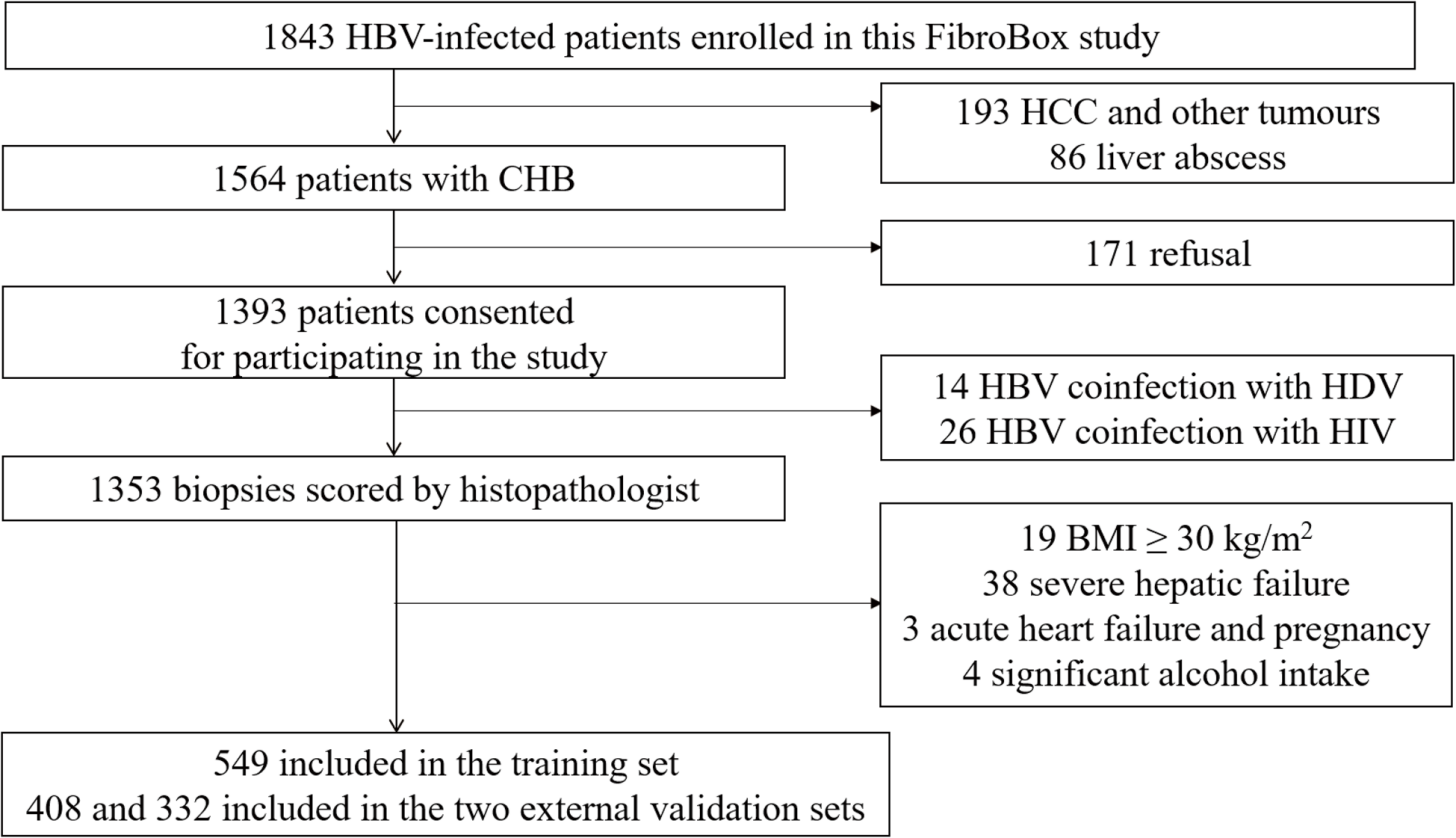
Flow diagram of the study population and reasons for exclusion. CHB, chronic HBV; HCC, hepatocellular carcinoma; HDV, hepatitis D virus

Between July 2008 and November 2017, 1843 HBV-infected patients were retrospectively enrolled in this study (Fig. 1). After exclusion of patients with HCC or other tumors (*n* = 193) and liver abscess (*n* = 86), histological specimens of 1393 (75.6%) patients showed eligibility. A total of 171 (9.3%) patients refused to participate in this study. After the investigation of clinical information, 14 patients were found co-infected with HDV and 26 with HIV (Fig. 1). The data of 64 patients were incomplete. Therefore, 1289 patients were finally included in the study. The TE results of all the included patients were reliable according to guidelines proposed by Boursier et al. [16]. The main characteristics of the study patients are summarized in Table 1.

**Table 1:** Baseline characteristics of the study population in training set (Huai’an, Jilin and Anhui) and in validation sets (Beijing)

Variables	Huai’an (*n* = 252)	Jilin (*n* = 297)	Anhui (*n* = 408)	Beijing (*n* = 332)	*p* Value
Male sex, n	158 (63%)	168 (57%)	257 (63%)	203 (61%)	0.274
Median age (years) (IQR)	42 (32–49)	42 (33–50)	38 (29–48)	38 (30–47)	0.006
Median BMI (kg/m^2^) (IQR)	23.7 (22.5–25.1)	23.7 (21.6–26.0)	22.8 (20.4–25.4)	23.7 (21.0–26.1)	0.014
Median fasting LSM value (kPa)	10.4 (6.6–15.3)	7.9 (5.9–11.8)	5.7 (4.2–7.5)	7.8 (5.6–12.4)	< 0.001
Median ALT (IU/L) (IQR)	42 (27–67)	39 (24–65)	49 (26–84.5)	46.2 (25.2–79.4)	0.046
Median AST (IU/L) (IQR)	35 (27–55)	32 (25–50)	34 (23–51)	33 (25–52)	0.108
Median GGT (IU/L) (IQR)	46 (24–98)	37 (21–79)	31.5 (21–73)	32 (19–60)	< 0.001
Median total bilirubin (IU/L) (IQR)	15.8 (11.9–21.0)	14.4 (11.6–21.2)	17.7 (13.0–23.1)	14.3 (11.4–20)	0.654
Median platelet counts (10^9^/L) (IQR)	165 (122–206)	179 (140–215)	161.5 (125–204)	186 (148–225)	0.148
Median APRI (IQR)	0.64 (0.41–1.35)	0.51 (0.33–0.84)	0.55 (0.34–0.92)	0.50 (0.30–0.76)	< 0.001
Median FIB-4 (IQR)	1.61 (0.99–2.87)	1.22 (0.82–1.95)	1.17 (0.74–2.04)	1.07 (0.73–1.69)	< 0.001
Ultrasound size of spleen (mm^2^) (IQR)	4410 (3866–5280)	3780 (2976–4710)	3468 (3120–3813)	3620 (2890–4560)	< 0.001
Ultrasound diameter of spleen vein (mm) (IQR)	6 (5.2–7)	7 (6.7–8)	9 (8–10)	7 (6–8)	< 0.001
Ultrasound diameter of portal vein (mm) (IQR)	11 (10–12)	12 (11–12)	11 (10–11)	11 (10–12)	< 0.001
Ultrasound velocity of portal vein (m/s) (IQR)	0.16 (0.14–0.19)	0.11 (0.10–0.12)	Not reported	Not reported	
Median size of liver biopsy (mm) (IQR)	15 (12–16)	16 (12–19)	15 (12–20)	16 (13–18)	< 0.001
Metavir fibrosis stage (F0/F1/F2/F3/F4)	21 (8.3%)/37 (14.7%)/46 (18.3%)/51 (20.2%)/97 (38.5%)	32 (10.8%)/77 (25.9%)/84 (28.3%)/44 (14.8%)/60 (20.2%)	10 (2.5%)/144 (35.3%)/129 (31.6%)/66 (16.2%)/59 (14.5%)	26 (7.9%)/127 (38.3%)/73 (22%)/32 (9.6%)/74 (22.3%)	< 0.001
Metavir activity grade (A0/A1/A2/A3)	50 (19.8%)/106 (42.1%)/72 (28.6%)/24 (9.5%)	190 (64.0%)/54 (18.2%)/52 (17.5%)/1 (0.3%)	86 (21.1%)/254 (62.2%)/61 (15.0%)/7 (1.7%)	13 (3.9%)/154 (46.4%)/114 (34.3%)/51 (15.4%)	< 0.001

*ALT* alanine transaminase, *APRI* (AST)-to-platelet ratio index, *AST* aspartate transaminase, *BMI* body mass index, *GGT* gamma-glutamyl transpeptidase, *LSM* liver stiffness measurement

### Histopathology

No complication was reported after liver biopsy. The significant fibrosis and cirrhosis account for 63.2% (815) and 22.5% (290) of all included patients, respectively. Almost a quarter of patients (382; 29.6%) had liver activity (A2/A3) and no steatosis was reported by the histopathologists. Meanwhile, 994 (77.1%) specimens showed consistent results rendered by 2 pathologists and a final determined diagnosis was reached by a third experienced histopathologist for the remaining specimens that showed biases.

### Training sets in Huai’an and Jilin

**Fig. 2:**
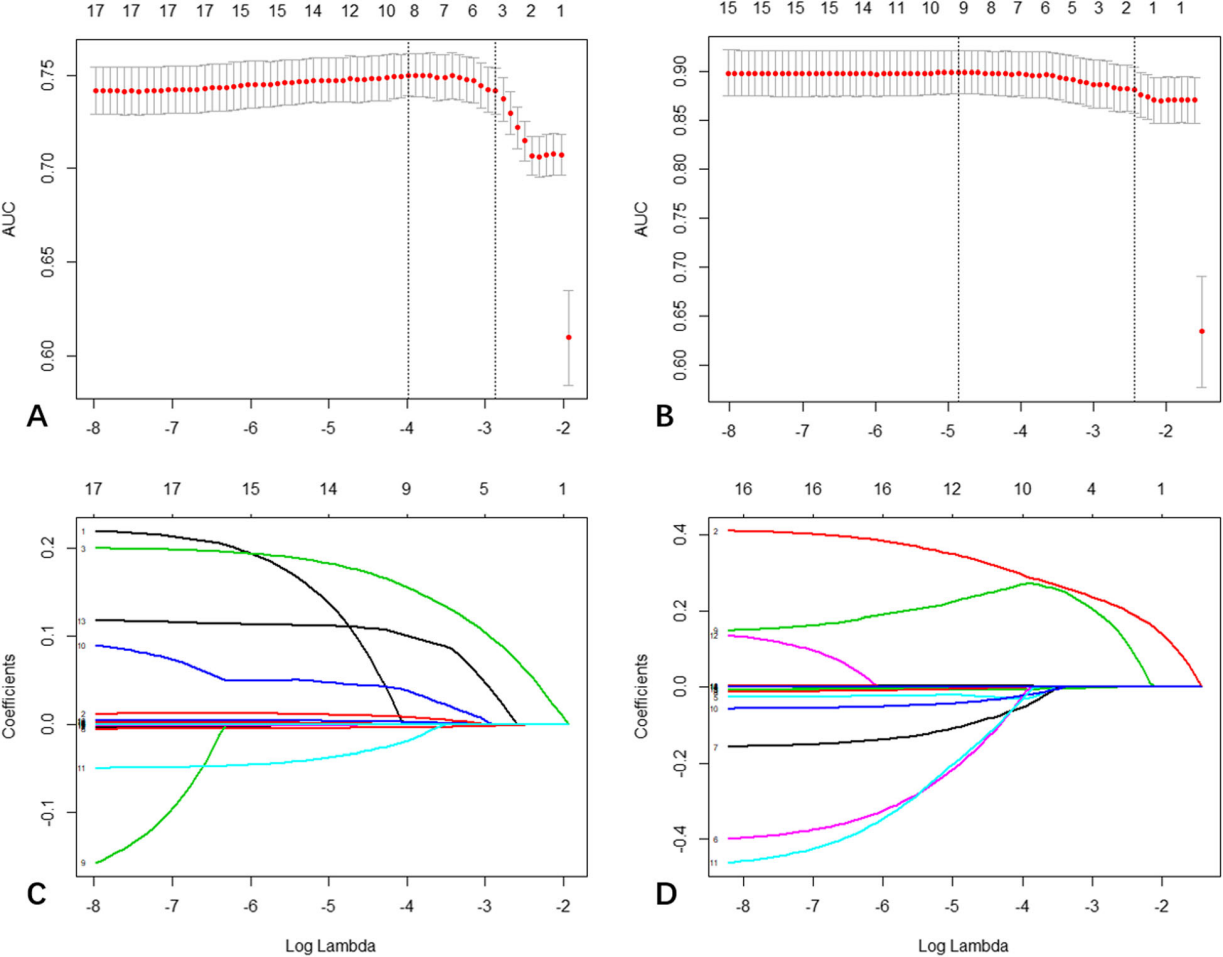
Feature selection by using a parametric method, the least absolute shrinkage and selection operator (LASSO) regression. **a** Significant fibrosis feature selection of tuning parameter (λ) in the LASSO model used 10-fold cross-validation via minimum criteria. The AUC curve was plotted versus log(λ). Dotted vertical lines were drawn at the optimal values by using the minimum criteria and the 1 standard error of the minimum criteria (the 1 – standard error criteria). The optimal log(λ) of − 3.96 was chosen. **b** Cirrhosis feature selection and the optimal log(λ) of − 4.83 was chosen. **c** LASSO coefficient profiles of the 18 initially selected features. A vertical line was plotted at the optimal λ value, which resulted in 9 features with nonzero coefficients. **d** LASSO coefficient profiles of the 16 initially selected features. A vertical line was plotted at the optimal λ value, which resulted in 9 features with nonzero coefficients

In spearman correlation analyses of original variables, the stage of liver fibrosis was associated with age, AST, GGT, total bilirubin, platelet count, WBC, PT, ALP, albumin, INR, PIIINP, type IV collagen, laminin, HA, size of spleen, diameter of spleen vein, diameter of portal vein, velocity of portal vein and Fibroscan results (Table 2). Subsequent multivariable analysis using the least absolute shrinkage and selection operator (LASSO) logistic regression (Fig. 2) and the filter method [20] (supplement material 1) selected Fibroscan results, platelet count, AST, PT, PIIINP, type IV collagen, laminin, HA and diameter of portal vein as input parameters of diagnostic models for significant fibrosis and cirrhosis.

**Table 2:** Selection for orginal variables associated with the presence of fibrosis stage in the training set

Variables	Spearman correlation analysis		Combined multivariate analysis	
	*r* value	*p* value	Significant fibrosis vs none	Cirrhosis vs F0–3
Age (years)	0.223	< 0.001		
Male sex, n (%)	−0.021	0.630		
BMI	−0.013	0.770		
ALT (IU/L)	0.030	0.489		
AST (IU/L)	0.171	< 0.001	√	√
GGT (IU/L)	0.187	< 0.001	√	√
Total bilirubin (IU/L)	0.110	0.010		
Platelet count (10^9^/L)	−0.356	< 0.001	√	√
WBC (10^9^/L)	−0.257	< 0.001		
PT (s)	0.321	< 0.001	√	√
ALP (IU/L)	0.094	0.027		
Albumin (g/L)	−0.144	0.001		
Cholesterol (mmol/L)	−0.071	0.099		
INR	0.356	< 0.001		
PIIINP (ng/ml)	0.330	< 0.001	√	√
Type IV collagen (ng/ml)	0.478	< 0.001	√	√
Laminin (ng/ml)	0.465	< 0.001	√	√
HA (ng/ml)	0.444	< 0.001	√	√
Ultrasound size of spleen (mm^2^)	0.223	< 0.001		
Ultrasound diameter of spleen vein (mm)	0.097	0.024		
Ultrasound diameter of portal vein (mm)	0.138	0.001	√	√
Ultrasound velocity of portal vein (m/s)	0.179	< 0.001		
Fibroscan results (kPa)	0.767	< 0.001	√	√

*ALT* alanine transaminase, *ALP* alkaline phosphatase, *AST* aspartate transaminase, *BMI* body mass index, *GGT* gamma-glutamyl transpeptidase, *HA* hyaluronic acid, *INR* international normalized ratio, *PIIINP* type III procollagen aminoterminal peptide, *PT* prothrombin time, *WBC* white blood cell

In the training cohort, the AUROC of the FibroBox for predicting significant fibrosis (0.914, 95% CI 0.890 to 0.938) was higher than that of the models using TE alone (0.886, 95% CI 0.856 to 0.917), APRI (0.692, 95% CI 0.643 to 0.741) or FIB-4 (0.707, 95% CI 0.659 to 0.755). The optimal cut-off value of FibroBox was 0.38.

For predicting cirrhosis, the AUROC of FibroBox (0.914, 95% CI 0.885 to 0.943) was better than that of TE (0.880, 95% CI 0.844 to 0.917), APRI (0.705, 95% CI 0.659 to 0.752) and FIB-4 (0.758, 95% CI 0.713 to 0.804). The optimal cut-off value of FibroBox was 0.56.

### Validation set in Anhui

In the Anhui cohort (*n* = 408), fibrosis stage based on histopathology was shown as follows: 10 (2.5%) in F0, 144 (35.3%) in F1, 129 (31.6%) in F2, 66 (16.2%) in F3 and 59 (14.5%) in F4 (Table 1).

**Fig. 3:**
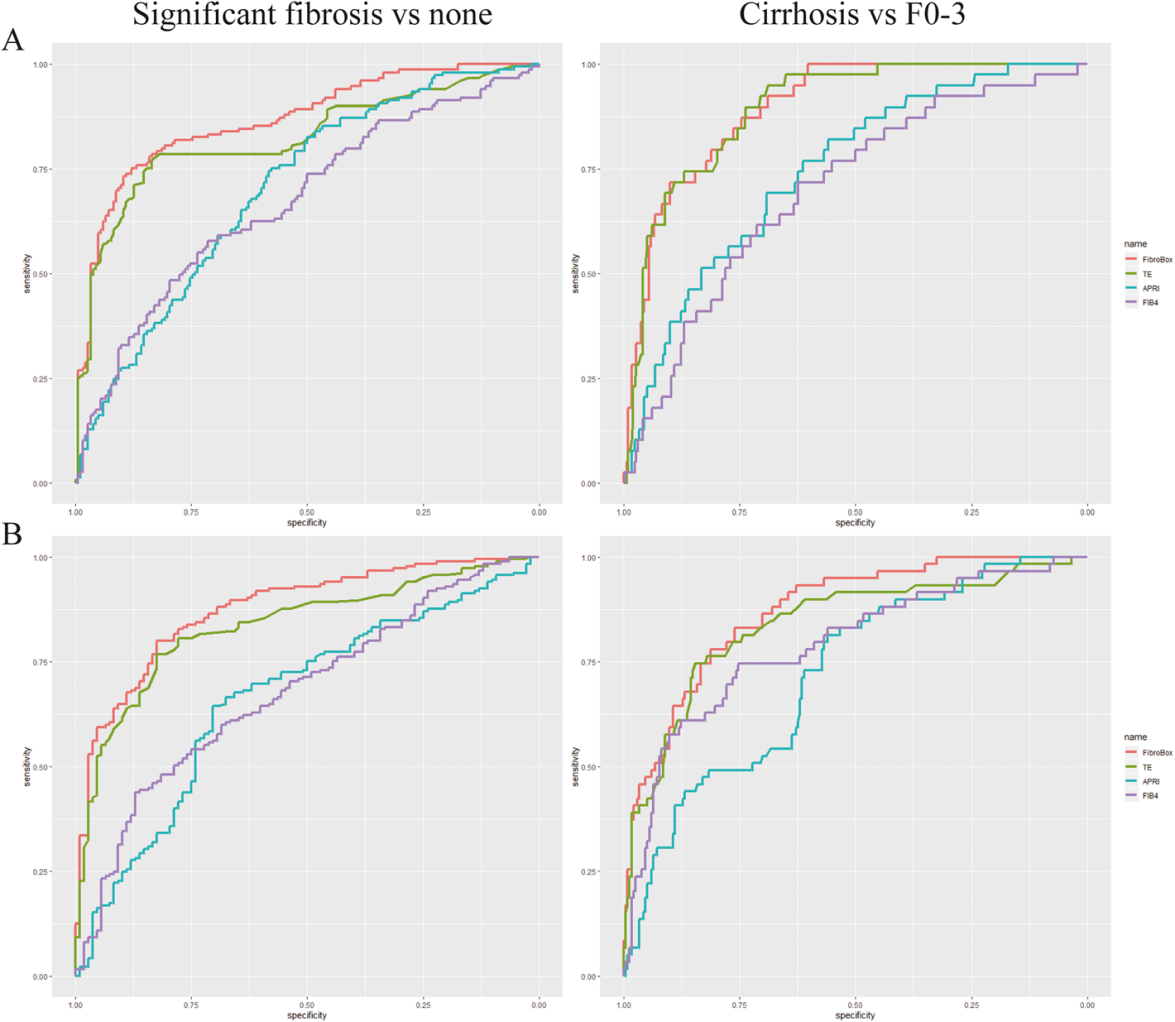
The performances of the prediction models including FibroBox, TE, APRI and FIB-4 for significant fibrosis and cirrhosis in the Anhui cohort (**a**) and Beijing corhort (**b**) are assessed by the area under a receiver operating characteristic (ROC) curve

The diagnostic performance (Fig. 3a) of FibroBox was better than TE, APRI and FIB-4: AUROC at 0.88 (95% CI 0.84 to 0.92) for predicting significant fibrosis and 0.87 (95% CI 0.82 to 0.92) for predicting cirrhosis (Table 3). Applying the optimal cut-off value (0.38 for significant fibrosis and 0.56 for cirrhosis) determined in the training set, the correctly classified rate of predicting significant fibrosis and cirrhosis was both 0.81 (se: 0.80, sp.: 0.82; se: 0.51, sp.: 0.94, respectively).

**Table 3:** Diagnostic performance of FibroBox, TE, APRI and FIB-4 in the validation cohorts (Anhui and Beijing)

Validation cohorts	Anhui (***n*** = 408)		Beijing (***n*** = 332)	
	F0–1 vs F2–4	F0–3 vs F4	F0–1 vs F2–4	F0–3 vs F4
**FibroBox**				
AUROC (95% CI)	0.88 (0.84 to 0.92)	0.87 (0.82 to 0.92)	0.87 (0.83 to 0.91)	0.90 (0.85 to 0.94)
Cut-off values	0.38	0.56	0.38	0.56
Sensitivity/specificity (%)	80/82	78/81	75/88	72/90
Correctly classified (%)	81	81	82	88
PPV/NPV (%)	89/71	51/94	84/81	49/96
Positive/negative LR	4.5/0.2	4.1/0.3	6.2/0.3	7.2/0.3
**TE**				
AUROC (95% CI)	0.84 (0.79 to 0.88)	0.84 (0.78 to 0.90)	0.82 (0.77 to 0.87)	0.89 (0.85 to 0.94)
Cut-off values	7.8	11.3	7.8	11.3
Sensitivity/specificity (%)	77/82	75/85	77/84	95/69
Correctly classified (%)	79	83	81	72
PPV/NPV (%)	88/67	55/93	79/82	29/99
Positive/negative LR	4.4/0.3	4.8/0.3	4.7/0.3	3.0/0.1
**APRI**				
AUROC (95% CI)	0.66 (0.60 to 0.73)	0.72 (0.65 to 0.79)	0.70 (0.65 to 0.76)	0.75 (0.67 to 0.82)
Cut-off values	0.50	0.50	0.43	0.62
Sensitivity/specificity (%)	64/70	81/56	75/58	69/69
Correctly classified (%)	67	61	66	69
PPV/NPV (%)	79/54	32/92	59/74	23/94
Positive/negative LR	2.2/0.5	1.8/0.3	1.8/0.4	2.2/0.4
**FIB-4**				
AUROC (95% CI)	0.68 (0.62 to 0.74)	0.79 (0.72 to 0.86)	0.67 (0.61 to 0.73)	0.70 (0.62 to 0.79)
Cut-off values	1.71	1.62	1.20	1.20
Sensitivity/specificity (%)	44/87	75/75	58/71	71/62
Correctly classified (%)	60	75	65	63
PPV/NPV (%)	85/47	43/92	62/67	20/94
Positive/negative LR	3.4/0.6	3.0/0.3	2.0/0.6	1.9/0.5
**Comparison of AUROC**				
FibroBox and TE	< 0.001	0.058	< 0.001	0.863
FibroBox and APRI	< 0.001	< 0.001	< 0.001	< 0.001
FibroBox and FIB-4	< 0.001	0.015	< 0.001	< 0.001
TE and APRI	< 0.001	0.007	< 0.001	< 0.001
TE and FIB-4	< 0.001	0.264	< 0.001	< 0.001
APRI and FIB-4	0.575	0.009	0.264	0.211

*APRI* AST-to-platelet ratio index, *AST* aspartate transaminase, *AUROC* area under the receiver operating characteristic curve, *LR* likelihood ratio, *NPV* negative predictive value, *PPV* positive predictive value, *TE* transient elastography

**Fig. 4:**
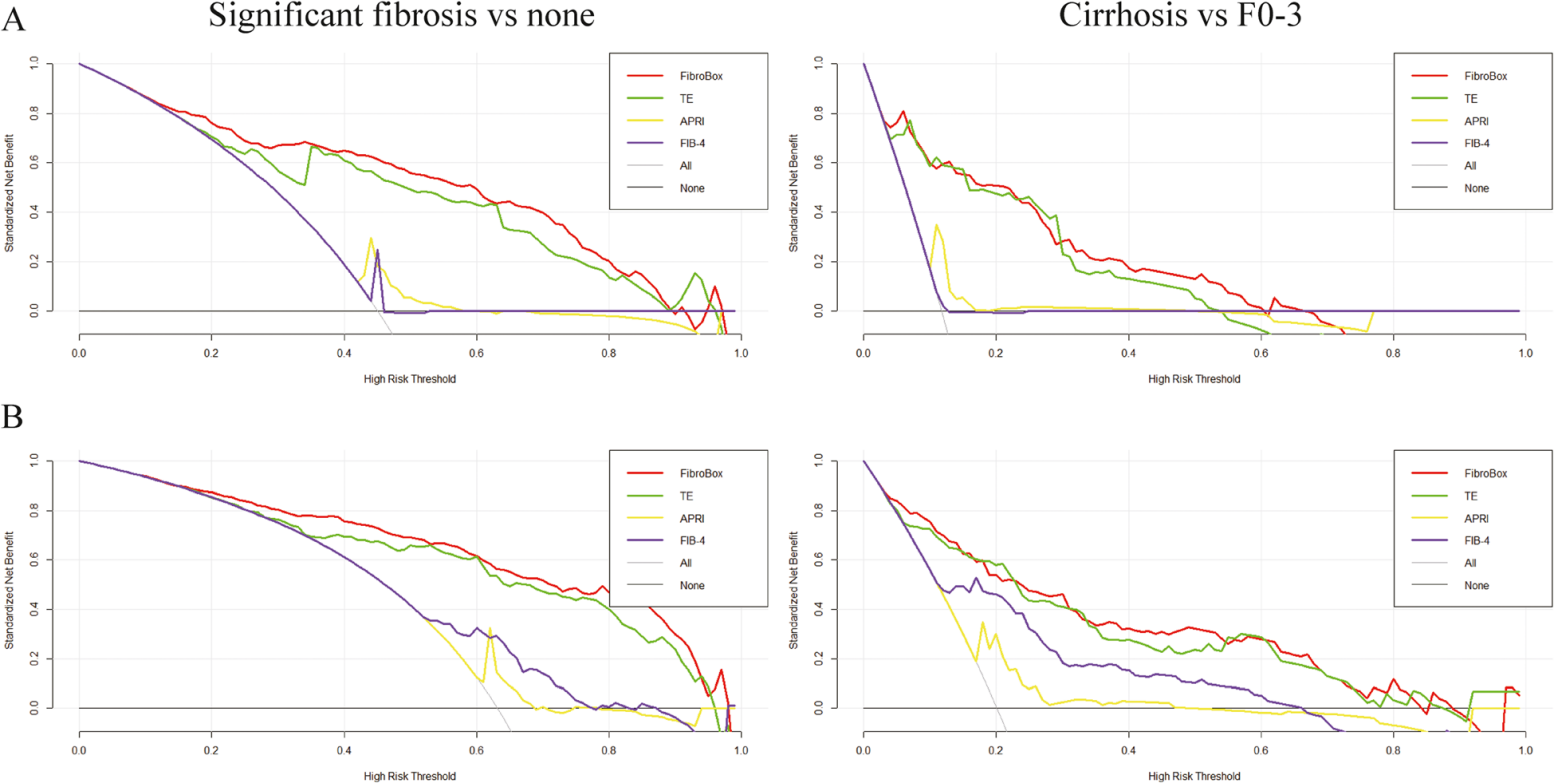
Decision curve analysis (DCA) of the prediction models including FibroBox, TE, APRI and FIB-4 for significant fibrosis and cirrhosis in the Anhui cohort (**a**) and Beijing corhort (**b**)

Across the range of reasonable threshold probabilities in this cohort, DCA graphically demonstrated that FibroBox provided a larger net benefit compared with TE, APRI and FIB-4 in diagnosing significant fibrosis and cirrhosis (Fig. 4a). This became as the supplementary evidence for the comparison of FibroBox and TE (*p* = 0.058) in predicting cirrhosis.

### Validation set in Beijing

In the Beijing cohort (*n* = 332), 26 (7.9%) were F0, 127 (38.3%) were F1, 73 (22%) were F2, 32 (9.6%) were F3 and 74 (22.3%) were F4 according to the liver histology results (Table 1).

For the prediction of significant fibrosis (Fig. 3b), it was statistically significant that the AUROC of FibroBox (0.87, 95% CI 0.83 to 0.91) was higher than that of TE (0.82, 95% CI 0.77 to 0.87, *p* <  0.001), APRI (0.70, 95% CI 0.65 to 0.76, *p* <  0.001) and FIB-4 (0.67, 95% CI 0.61 to 0.73, *p* <  0.001) (Table 3). For predicting cirrhosis (Fig. 3b), the performance of FibroBox (0.90, 95% CI 0.85 to 0.94) was significantly better than that of APRI (0.75, 95% CI 0.67 to 0.82, *p* <  0.001) and FIB-4 (0.70, 95% CI 0.62 to 0.79, *p* <  0.001) (Table 3). There was no significant difference between FibroBox and TE (0.89, 95% CI 0.85 to 0.94, *p* = 0.863). DCA also showed consistent results (Fig. 4b).

## Discussion

In China, assessing the severity of CHB infection is a critical step before timely intervention [4]. TE has also been widely applied in Chinese hospitals in recent years, regardless of its high price.

To stage liver fibrosis noninvasively in patients with HBV, our study established and validated a multivariable model based on machine-learning and incorporating Fibroscan results, serum biomarker indices and ultrasonic measurements. This FibroBox model demonstrated favorable diagnostic performances in two external validation cohorts for the prediction of significant fibrosis which was superior to TE, APRI and FIB-4. The diagnostic performance of FibroBox for predicting cirrhosis was potentially better than TE, which required more validations.

It was reported that Fibroscan performed better than serum biomarker indexes in predicting significant fibrosis and cirrhosis in Chinese cohorts [21, 22]. In our study, TE measurements were obtained within a month after liver biopsy. The optimal cut-off values of Fibroscan for significant fibrosis and cirrhosis in both validation sets were 7.8 and 11.3 kpa, both close to those proposed in other countries [23–25]. Regardless of set types and prediction goals, all the AUROC results of TE were over 0.8, which was acceptable but not efficient enough. Our study excluded obese patients (BMI ≥30 kg/m2), thus ruling out an error leading to unreliable TE results. Fibroscan is not widespread because of its high cost (€34,000 for a portable device and €5000 for its annual maintenance), but its high diagnostic efficiency also makes it recommendable [5, 26]. FibroBox behaved better than TE according to AUROC comparisons (Table 3, Fig. 3) and DCA curves (Fig. 4). Although the difference between FibroBox and TE for cirrhosis is not significant, the imbalance of data can also affect the validation results. For instance, less than a quarter of included patients were cirrhotic (Anhui: 14.5%; Beijing: 22.3%).

The application of Fibroscan is limited by ascites and not so reliable compared as two-dimensional (2D) shear wave elastography (SWE) [27, 28]. However, 2D-SWE has not been widely applied like Fibroscan in China. Therefore, this study took TE as the only input variable. In addition, TE has the advantage of staging liver fibrosis regardless of causes (HBV, HCV or nonalcoholic fatty liver disease [NAFLD]). FibroBox only focused on the HBV-induced liver fibrosis, which required more similar studies about other kinds of fibrosis.

The prediction accuracy of APRI and FIB-4 observed in this study was unacceptable. The AUROC of APRI was 0.66 (0.60 to 0.73) in the Anhui cohort and 0.70 (0.65 to 0.76) in the Beijing cohort in predicting significant fibrosis, and 0.72 (0.65 to 0.79) in the Anhui cohort and 0.75 (0.67 to 0.82) in the Beijing cohort in predicting cirrhosis. The diagnostic performance of APRI in the prediction of cirrhosis was better than that of which in the prediction of significant fibrosis. The AUROC value of FIB-4 in predicting cirrhosis in the Anhui cohort was significantly higher than that of APRI (*P* = 0.009), indicating FIB-4 might have a prediction efficiency between those of APRI and TE. In addition, the optimal cut-off values of APRI and FIB-4 were both calculated with Youden index (sensitivity + specificity - 1), and the optimal cut-off value of APRI was quite different from that recommended by the WHO guidelines [29], reminding of the instability and unreliability of APRI guideline-suggested cutoff values for the prediction of fibrosis in Chinese cohort.

There are several limitations in this study. First, the robustness of data was limited because of the retrospective researches. However, the size of research data is large and four centers participated in this study which can ensure the applicability and reliability of established models. We designed a two-validation-set study similar to that conducted by Lemoine et al. [25]. Second, the data sample inconsistency affected the model validations. For instance, the proportion of cirrhosis was only 14.5% in Anhui cohort, meaning that it cannot be taken as a training set, because this proportion is not enough to discriminate cirrhosis (F4) from non-cirhosis (F0–3). Third, the FibroBox is complicated and involves 10 parameters. However, the cost-effectiveness of this might not be poor because these 10 input parameters can be obtained through clinical examinations and the run time of FibroBox is only a few seconds. Finally, several parameters such as PIIINP, type IV collagen, laminin and HA are not readily available in clinical laboratories. We can develop several easily obtained ratios similar to the study conducted by Yuan et al. [30]. Future versions of FibroBox should focus on the simplification with accuracy.

## Conclusions

In conclusion, compared with TE, APRI and FIB-4, FibroBox may be a superior noninvasive fibrosis indicator to predict the fibrosis stage in Chinese patients with CHB. The FibroBox requires further validation in other parts of China or other countries.

## Supplementary information

**Additional file 1.**

## Data Availability

The data is not available because of patients’ privacy.

## Abbreviations

ALP: Alkaline phosphatase
ALT: Alanine transaminase
APRI: Aspartate transaminase-to-platelet ratio index
AST: Aspartic transaminase
AUROC: Area under the receiver operating characteristic curve
BMI: Body mass index
CHB: Chronic hepatitis B
DCA: Decision curve analysis
FIB-4: Fibrosis-4
GGT: Gamma-glutamyl transpeptidase
HA: Hyaluronic acid
HBsAg: Hepatitis B surface antigen
HBV: Hepatitis B virus
HCC: Hepatocellular carcinoma
HCV: Hepatitis C virus
HDV: Hepatitis D virus
HIV: Human immunodeficiency virus
INR: International normalized ratio
kPa: kilopascal
LB: Liver biopsy
LSMs: Liver stiffness measurements
MRE: Magnetic resonance elastography
PIIINP: Type III procollagen aminoterminal peptide
PT: Prothrombin time
TE: Transient elastography
ULN: The upper limit of normal
WBC: White blood cell
2D-SWE: Two-dimensional shear wave elastography

## References

[1] Seto WK, Lo YR, Pawlotsky JM, Yuen MF (2018). Chronic hepatitis B virus infection. Lancet..

[2] Schweitzer A, Horn J, Mikolajczyk RT, Krause G, Ott JJ (2015). Estimations of worldwide prevalence of chronic hepatitis B virus infection: a systematic review of data published between 1965 and 2013. Lancet..

[3] Ott JJ, Horn J, Krause G, Mikolajczyk RT (2017). Time trends of chronic HBV infection over prior decades - a global analysis. J Hepatol.

[4] Wang FS, Fan JG, Zhang Z, Gao B, Wang HY (2014). The global burden of liver disease: the major impact of China. Hepatology..

[5] European Association for the Study of the Liver (2017). EASL 2017 clinical practice guidelines on the management of hepatitis B virus infection. J Hepatol.

[6] Rockey DC, Caldwell SH, Goodman ZD, Nelson RC (2009). Smith AD; American Association for the Study of Liver Diseases. Liver Biopsy Hepatology.

[7] Perrault J, McGill DB, Ott BJ, Taylor WF (1978). Liver biopsy: complications in 1000 inpatients and outpatients. Gastroenterology..

[8] Strassburg CP, Manns MP (2006). Approaches to liver biopsy techniques--revisited. Semin Liver Dis.

[9] Maharaj B, Maharaj RJ, Leary WP, Cooppan RM, Naran AD, Pirie D, Pudifin DJ (1986). Sampling variability and its influence on the diagnostic yield of percutaneous needle biopsy of the liver. Lancet..

[10] Regev A, Berho M, Jeffers LJ, Milikowski C, Molina EG, Pyrsopoulos NT, Feng ZZ, Reddy KR, Schiff ER (2002). Sampling error and intraobserver variation in liver biopsy in patients with chronic HCV infection. Am J Gastroenterol.

[11] European Association for Study of Liver; Asociacion Latinoamericana para el Estudio del Higado (2015). EASL-ALEH clinical practice guidelines: non-invasive tests for evaluation of liver disease severity and prognosis. J Hepatol.

[12] Obermeyer Z, Emanuel EJ (2016). Predicting the future - big data, machine learning, and clinical medicine. N Engl J Med.

[13] Chen JH, Asch SM (2017). Machine learning and prediction in medicine - beyond the peak of inflated expectations. N Engl J Med.

[14] Bedossa P, Poynard T (1996). An algorithm for the grading of activity in chronic hepatitis C. The METAVIR Cooperative Study Group. Hepatology.

[15] Sandrin L, Fourquet B, Hasquenoph JM, Yon S, Fournier C, Mal F, Christidis C, Ziol M, Poulet B, Kazemi F, Beaugrand M, Palau R (2003). Transient elastography: a new noninvasive method for assessment of hepatic fibrosis. Ultrasound Med Biol.

[16] Boursier J, Zarski JP, de Ledinghen V, Rousselet MC, Sturm N, Lebail B, Fouchard-Hubert I, Gallois Y, Oberti F, Bertrais S, Calès P, Multicentric Group from ANRS/HC/EP23 FIBROSTAR Studies (2013). Determination of reliability criteria for liver stiffness evaluation by transient elastography. Hepatology..

[17] Wai CT, Greenson JK, Fontana RJ, Kalbfleisch JD, Marrero JA, Conjeevaram HS, Lok AS (2003). A simple noninvasive index can predict both significant fibrosis and cirrhosis in patients with chronic hepatitis C. Hepatology..

[18] Vallet-Pichard A, Mallet V, Pol S (2006). FIB-4: a simple, inexpensive and accurate marker of fibrosis in HCV-infected patients. Hepatology..

[19] Delong ER, Clarke-Pearson DLL (1988). Comparing the areas under two or more correlated receiver operating characteristic curves: a nonparametric approach. Biometrics.

[20] Torlay L, Perrone-Bertolotti M, Thomas E, Baciu M (2017). Machine learning-XGBoost analysis of language networks to classify patients with epilepsy. Brain Inform.

[21] Chen Y, Wang Y, Chen Y, Yu Z, Chi X, Hu KQ, Li Q, Tan L, Xiang D, Shang Q, Lei C, Chen L, Hu X, Wang J, Liu H, Lu W, Chi W, Dong Z, Wang X, Li Z, Xiao H, Chen D, Bai W, Zhang C, Xiao G, Qi X, Chen J, Zhou L, Sun H, Deng M, Qi X, Zhang Z, Qi X, Yang Y (2019). A novel noninvasive program for staging liver fibrosis in untreated patients with chronic hepatitis B. Clin Transl Gastroenterol.

[22] Lu XJ, Li XH, Yuan ZX, Sun HY, Wang XC, Qi X, Zhang X, Sun B (2018). Assessment of liver fibrosis with the gamma-glutamyl transpeptidase to platelet ratio: a multicentre validation in patients with HBV infection. Gut..

[23] Marcellin P, Ziol M, Bedossa P, Douvin C, Poupon R, de Lédinghen V, Beaugrand M (2009). Non-invasive assessment of liver fibrosis by stiffness measurement in patients with chronic hepatitis B. Liver Int.

[24] Castéra L, Bernard PH, Le Bail B, Foucher J, Trimoulet P, Merrouche W, Couzigou P, de Lédinghen V (2011). Transient elastography and biomarkers for liver fibrosis assessment and follow-up of inactive hepatitis B carriers. Aliment Pharmacol Ther.

[25] Lemoine M, Shimakawa Y, Nayagam S, Khalil M, Suso P, Lloyd J, Goldin R, Njai HF, Ndow G, Taal M, Cooke G, D'Alessandro U, Vray M, Mbaye PS, Njie R, Mallet V, Thursz M (2016). The gamma-glutamyl transpeptidase to platelet ratio (GPR) predicts significant liver fibrosis and cirrhosis in patients with chronic HBV infection in West Africa. Gut..

[26] Terrault NA, Lok ASF, McMahon BJ, Chang KM, Hwang JP, Jonas MM, Brown RS, Bzowej NH, Wong JB (2018). Update on prevention, diagnosis, and treatment of chronic hepatitis B: AASLD 2018 hepatitis B guidance. Hepatology..

[27] Dietrich CF, Bamber J, Berzigotti A, Bota S, Cantisani V, Castera L, Cosgrove D, Ferraioli G, Friedrich-Rust M, Gilja OH, Goertz RS, Karlas T, de Knegt R, de Ledinghen V, Piscaglia F, Procopet B, Saftoiu A, Sidhu PS, Sporea I, Thiele M (2017). EFSUMB guidelines and recommendations on the clinical use of liver ultrasound Elastography, update 2017 (long version). Ultraschall Med.

[28] Jeong JY, Cho YS, Sohn JH (2018). Role of two-dimensional shear wave elastography in chronic liver diseases: a narrative review. World J Gastroenterol.

[29] WHO. World Health Organization (2015). Guidelines for the Prevention, Care and Treatment of Persons with chronic Hepatitis B infection.

[30] Yuan X, Duan SZ, Cao J, Gao N, Xu J, Zhang L (2019). Noninvasive inflammatory markers for assessing liver fibrosis stage in autoimmune hepatitis patients. Eur J Gastroenterol Hepatol.

