# Supplementary material for: FibroBox: a novel noninvasive tool for predicting significant liver fibrosis and cirrhosis in HBV infected patients
Source: Biomark Res. 2020 Sep 25;8:48. doi: 10.1186/s40364-020-00215-2 (PMC7520974; doi:10.1186/s40364-020-00215-2)
Supplement: Supplementary file 1 — Additional file 1. [file 40364_2020_215_MOESM1_ESM.docx]

**Supplement Material 1**

1. **Data preprocessing**

The main purpose of the algorithm flow is to select the significant features, design the classifier and establish the predictive model by means of supervised learning. According to the METAVIR scoring system, the liver fibrosis can be staged into F0-F4. In this study, we generated two kinds of labels (named as Label A and Label B) based on fibrosis stages. For Label A, we describe positive and negative samples at the threshold of F1 (non-significant fibrosis vs. significant fibrosis). For Label B, we set the threshold as F3 (non-cirrhosis vs. cirrhosis).

We extract the stages(F0-F4) of METAVIR scoring system as our labels, while there exist null values and some abnormal conditions in the corresponding field, we take the following exclusion criteria for preliminary data preprocessing: (1) absence of fibrosis staging; (2) incorrect format of staging records such as string (’ss’ as an example in our data source); (3) the null rate of a field is higher than 70%, while for other null values, we use the mean of the corresponding field. If several stages were recorded, the most advanced stage was recorded for this study [1]. For instance, if there is a record of F2-3, we maintain the stage as F3.

For character data field (like sex, pathogenesis), we deal it with the LabelEncoder method in Python. After series operations of null value dispose and data conversion, we then check for the existing outliers for other fields. As shown in Supplement figure 1, it’s a whisker plot for the outlier examinations while inputdata had been processed by above methods. Considering the dispersion of some serological targets, we can filter out the outliers. It is noteworthy that some outliers have clinical reference significance, such as the ultrasound size of spleen, we can see from the box plot that there exists some size higher than 5000. In this study, we reserved these values that seemd like outliers instead of discarding them to maintain a certain level of true scene.

1. **Methods**
   1. **Logistic Regression as Baseline**

In this study, we developed the logistic regression model as a baseline to verify the feature selection. Considering the classification task, a binary logistic model accepts input *x* and generate the predicted value *y*$\epsilon${0,1} by means of logistic function. We assumed the model function is parameterized by *θ*.

$$\sigma\left( x;\theta\right)=P\left( y=1 | x;\theta\right)=\frac{1}{1+e^{-\theta^{T}x}}$$

$$P\left( y=0 | x;\theta\right)=1-P\left( y=1 | x;\theta\right)$$

According to the maximum likelihood method, the objective loss function is defined as below:

$$L\left( \theta| x \right)=\prod_{i} P\left( y_{i} | x_{i};\theta\right)=\prod_{i} {\sigma(x;\theta)}^{y_{i}}{(1-\sigma x;\theta)}^{1-y_{i}}$$

The aim of this process is to minimize the function by training, and the model gradually learns to classify.

- 1. **XGBoost**

XGBoost [2] is an optimized distributed gradient boosting designed to be highly efficient, flexible and portable. It implements machine learning algorithms under the Gradient Boosting framework. Unlike traditional GBDT methods, XGBoost uses the Taylor binomial expansion to approximate the objective function, which supports a variety of objective functions including regression, classification and sorting.

- 1. **LightGBM**

LightGBM [3] is a gradient boosting framework using tree based learning algorithms. It is designed to be distributed and efficient. Compared with other models, it has many advantages such as faster training speed and higher efficiency, lower memory usage, better accuracy, support of parallel and GPU learning, capable of handling large-scale data. LightGBM uses the leaf-wise strategy to find a leaf with largest splitter gain unlike the inefficient leavel-wise strategy which generated redundant computing.

- 1. **Feature Processing**

Feature selection and feature extraction significantly contribute to feature engineering. In data collected from four centers, the common features were obtained, in which there are 24 dimensions in total: sex, age, BMI, fibroscan results, ALT, AST, GGT, Tbil, Plt, WBC, PTA, PT, ALP, albumin, cholesterol, INR, PIIINP, type IV collagen, laminin, HA, ultrasound spleen size, ultrasound diameter of spleen vein, ultrasound diameter of portal vein and ultrasound velocity of portal vein.

After fundamental data preprocessing such as normalization, significative features need to be selected from data fields, and then input them into algorithm model for machine learning. For feature selection, there are high-order statistics (e.g. lasso regression) and three classical machine learning methods including filter, wrapper and embedded [4]. In this study, we focused on the lasso regression and filter method. The main structure of lasso regressions has been shown in the manuscript. The main idea of the filter methods is to calculate the divergence of features and the correlation of between features and the labels. The features of low divergence and weak correlation were filtered out by setting the threshold. The procedure of filter methods can be divided into four ways. First, the variance corresponding to every feature was computed and the feature with variance value higher than threshold was selected. Next is the Pearson Correlation Coefficient which can only measure linear correlation. We need to calculate the correlation coefficient of each feature corresponding to the label and the P value (ranging from -1 to 1). The calculation formula is shown as follows:

$$p_{X, Y}=\frac{cov(X,Y)}{\sigma_{X}\sigma_{Y}}=\frac{E[(X-\mu_{X})(Y-\mu_{Y})]}{\sigma_{X}\sigma_{Y}}$$

The other two filter methods are chi-square test and mutual information. These four filter methods and the lasso regression were tried to compute four sets of features. Finally, 9 features were selected: Fibroscan results, platelet count, ALT, PT, PIIINP, type IV collagen, laminin, HA and diameter of spleen vein.

**2.5 Training and Validation**

We conducted experiments from four directions mainly, first we deal with table respectively, thenwe trained on data from different regions, next we

merged all tables to get more data. Lastly considered the data of Beijing is the most and comparatively balanced, we merge the remaining data as training data and regard the Beijing table as validation data. We do *k*-fold cross-validation for other three methods all the way(in our research, *k* = 5).

**3 Experiments**

For feature selection, we conduct series of heuristic search on logistic regression model as our baseline. And then, we tune parameters to get good results by means of grid search, random search and Hyperopt, considering XGBoost and LightGBM algorithms. We selected LightGBM algorithm as our final model and the learning rate was set to 0.1. The number of leaves are frequently set as 70 or 80 to get better accuracy, but 90 is the best choice in this study. To prevent overfitting, the index of colsample_bytree was set to 0.9. Besides, it can speed up training.

The classified ability of our models was scored by five common evaluation metrics, which are precision, recall, F1-score, accuracy and AUC respectively. There are four kinds of results of a binary classifier: True Positive(TP), False Positive(FP), True Negative(TN), False Negative(FN). Precision and Recall is set for the specific class, for example, the following
formulas are for positive samples:

$$Precision=\frac{TP}{TP+FP}$$

$$Recall=\frac{TP}{TP+FN}$$

In general, F1-score is used to combine precision and recall, the corresponding formula is shown below:

$$F1-score=\frac{2\times Precision\times Recall}{Precision+Recall}$$

The metric of accuracy is defined as:

$$Accuracy=\frac{TP+FN}{TP+TN+FP+FN}$$

The above metrics are calculated by probability and the selected threshold, while the setting of threshold has a great influence on the calculation. To solve the problem, in the same spirit of previous studies [1,4,5], classification performance was scored with the area under the receiver operating characteristic curve (AUC). The AUC can be interpreted as the probability of a positive instance is greater than a negative one [6]. For different label, the receiver operating curve (ROC) used the TP rate as the vertical axis while the FP rate as the horizontal axis for positive condition. Moreover, the AUC can be calculated as the area under the receiver operating characteristic curve, the value is positively correlated with the classifier performance.

**References**

[1] Koichiro Yasaka, Hiroyuki Akai, Akira Kunimatsu, Osamu Abe, and Shigeru Kiryu. Deep learning for staging liver fibrosis on ct: a pilot study. *European radiology*, 28(11):4578–4585, 2018.

[2] Tianqi Chen and Carlos Guestrin. Xgboost: A scalable tree boosting system. In *Proceedings of the 22nd acm sigkdd international conference on knowledge discovery and data mining*, pages 785–794. ACM, 2016.

[3] Guolin Ke, Qi Meng, Thomas Finley, Taifeng Wang, Wei Chen, Weidong Ma, Qiwei Ye, and Tie-Yan Liu. Lightgbm: A highly efficient gradient boosting decision tree. In *Advances in Neural Information Processing Systems*, pages 3146–3154, 2017.

[4] L Torlay, Marcela Perrone-Bertolotti, E Thomas, and Monica Baciu. Machine learning–xgboost analysis of language networks to classify patients with epilepsy. *Brain informatics*, 4(3):159, 2017.

[5] Cucchetti Alessandro, Piscaglia Fabio, Grigioni Antonia D’Errico, Ravaioli Matteo, Cescon Matteo, Zanello Matteo, Grazi Gian Luca, Golfieri Rita, Grigioni Walter Franco, and Pinna Antonio Daniele. Preoperative prediction of hepatocellular carcinoma tumour grade and micro-vascular invasion by means of artificial neural network: a pilot study. *Journal of Hepatology*, 52(6):880–888, 2010.

[6] Charles X. Ling, Huang Jin, and Harry Zhang. Auc:A better measure than accuracy in comparing learning algorithms. In *Canadian Society for Computational Studies of Intelligence Conference on Advances in Artificial Intelligence*, 2003.
